# Effect of Si Addition on Structure and Corrosion Resistance of FeCoNiCr High-Entropy Alloy Coating

**DOI:** 10.3390/ma18010072

**Published:** 2024-12-27

**Authors:** Wenqiang Li, Jie Lian, Dengfeng Wang, Suo Zhang, Chengfu Han, Zhenyu Du, Fushan Li

**Affiliations:** 1 School of Materials Science and Engineering, Zhengzhou University, Zhengzhou 450001, China; 2 Henan Coal Science Academy Wear-Resistant Technology Co., Ltd., Zhengzhou 450001, China; 3 School of Mechanical and Electrical Engineering & Intelligent Manufacturing, Henan Open University, Zhengzhou 450008, China

**Keywords:** laser cladding, high-entropy alloy, microstructure analysis, corrosion resistance, coating

## Abstract

In this study, Fe_60_Co_10−x_Ni_15_Cr_15_Si_x_ (x = 0, 4, and 8) powders were successfully prepared using the aerosol method and employed to produce high-entropy coatings on Q235 steel via laser cladding. The microstructure and phase composition of the coatings were analyzed using scanning electron microscopy, energy-dispersive X-ray spectroscopy, and X-ray diffraction. Corrosion resistance and potential were evaluated through electrochemical analysis and Kelvin probe force microscopy. The results show that the Fe_60_Co_10−x_Ni_15_Cr_15_Si_x_ coatings exhibit excellent metallurgical bonding with no visible porosity or cracks. The coating primarily consists of an FCC structure; however, as the Si content increases, the structure transitions to a mixed FCC + BCC phase. The addition of Si also refines the grain size in the alloy system. Electrochemical analysis reveals that the Si0 and Si4 coatings exhibit similar corrosion behavior, while the Si8 coating shows a significant drop in corrosion potential, reducing its corrosion resistance. As the Si content increases, grain refinement leads to more grain boundaries, but the corrosion resistance decreases due to the lower corrosion performance of Si compared to Co. Considering both cost and corrosion resistance, the Si4 coating offers a balance of low cost and excellent corrosion resistance.

## 1. Introduction

High-entropy alloys (HEAs), which leverage the principle of configurational entropy, challenge the traditional alloy design paradigm reliant on a single principal element. HEAs offer superior properties such as high hardness, excellent wear and corrosion resistance, superior thermal stability, and superior irradiation resistance compared to conventional alloys [1,2]. However, HEAs have some drawbacks, including higher costs due to the use of expensive elements such as niobium (Nb) [3], vanadium (V) [4], tungsten (W), nickel (Ni), titanium (Ti) [5], and cobalt (Co) [6]. The widespread application of HEAs is further hindered by the high costs associated with their casting-based production methods.

The durability and performance of materials in various environments largely depend on surface quality, functionality, and mechanical properties [7]. To improve surface properties, numerous surface modification techniques are employed, including laser cladding, thermal spraying, magnetron sputtering, and plasma transfer arc cladding. Among these, laser cladding (LC) has been extensively studied and widely applied due to its ability to achieve controlled heat-affected zones (HAZs) with minimal dilution [8,9,10,11,12,13,14]. The high cooling rates (10^4^–10^6^ °C/s) in laser cladding promote structure refinement and prevent component segregation, making HEA-based coatings increasingly popular. HEA coatings are widely used in engineering and industrial applications for erosion resistance, corrosion protection, and stress mitigation [15]. Consequently, LC has emerged as a promising method for fabricating high-quality coatings with exceptional properties while addressing factors like processing complexity and environmental protection.

Currently, numerous laser-cladded HEA systems have been developed and extensively investigated. Among these, FeCoNiCr medium-entropy alloys (MEAs) with a face-centered cubic (FCC) structure are widely used due to their excellent solid solution capability [16,17,18]. Although FeCoCrNi MEAs exhibit good ductility, their hardness and strength are often insufficient for specific surface modification applications.

Recent studies have demonstrated that adding elements such as Mo, Al, Ti, Mn, and W to FeCoNiCr MEAs can improve their corrosion and wear resistance [19,20,21,22,23]. In addition, elements such as silicon (Si), chromium (Cr), nickel (Ni), and molybdenum (Mo) are frequently added to regulate phase structure refinement and enhance corrosion resistance in HEAs. These elements often contribute to passivation film formation, improving corrosion resistance. For example, the addition of Si element influences the thermodynamic state and structure of FeCoNiCr systems, as the approximate structure of this HEA system can be predicted based on its basic alloy parameters, including solution interaction (Ω), atomic size difference (δ), and valence electron concentration (VEC) [24,25]. The valence electron concentration of an equimolar FeCoCrNi system was calculated to be 8.25 (FCC structure) [26]. Yang et al. [27] reported that a solid solution is favored if δ < 6% and Ω > 1. Si element in the FCC phase of high-entropy alloys can enhance solid solution strengthening and reduce the size of the grain, resulting in an increase in the strength and toughness of the alloy [28,29]. The presence of Si element in the HEAs has been observed to enhance oxidation resistance by facilitating the formation of protective oxide layers on the surface, rendering them highly suitable for high-temperature applications [30].

Current research focuses on the phase transformation mechanisms of FeCoNiCr MEAs during heat treatment and deformation, with limited exploration of their low-cost application as coatings for corrosion resistance. Si element had a significant effect on the increase in the microhardness and wear resistance in FeCoCrNiSi HEAs, which is beneficial for its application in coating [31]. Fe_60_Co_15_Ni_15_Cr_10_ and Fe_60_Ni_15_Cr_15_Co_10_ medium-entropy alloys are composed primarily of a near-single FCC phase, which exhibits excellent plasticity, relatively low strength or hardness [16,32]. In order to reduce the raw material cost of these Fe-rich medium-entropy alloys and enhance their hardness, Si elements are added to this alloy system. In this study, Fe_60_Co_10−x_Ni_15_Cr_15_Si_x_ (x = 0, 4, and 8) high-entropy alloys were prepared based on these FeCoNiCr systems. Laser cladding technology was used to create coatings on Q235 steel, aiming to investigate the structural and corrosion resistance mechanisms. This study seeks to develop a cost-effective HEA coating with high corrosion resistance, with the goal of contributing to the successful application of HEAs.

## 2. Experimental Section

### 2.1. Sample Preparation

In this study, the Q235 alloy (provided by Zhongnuo New Material (Beijing) Technology Co., Ltd., Beijing, China, composition shown in Table 1) was used as the base material, and Fe, Co, Ni, Cr, and Si with 99.9% purity (provided by Zhongnuo New Material (Beijing) Technology Co., Ltd., purity, shape, and size shown in Table 2) were selected to prepare the alloy composition Fe_60_Co_10−x_Ni_15_Cr_15_Si_x_ (x = 0, 4, and 8). The alloy was melted in a vacuum arc furnace (Model: WK II, Physcience Opto-electronics Co., Ltd., Beijing, China), and 10 mm × 10 mm × 100 mm square columns were formed via suction casting. The specific procedure involves placing the raw materials, which have been formulated according to the nominal composition specified in the design, into the crucible of the copper mold within the vacuum arc furnace, followed by evacuation of the chamber. Once the vacuum level within the chamber reaches below 5 × 10^−3^ Pa, high-purity (99.9%) argon gas is introduced as a shielding medium. Subsequently, the high-purity titanium ingot is melted using the vacuum arc furnace to eliminate any residual oxygen present in the chamber. To ensure homogeneity of the alloy composition during the melting process, the alloy is remelted a minimum of five times.

Subsequently, Fe_60_Co_10−x_Ni_15_Cr_15_Si_x_ HEA powder was prepared using aero-chemical equipment (Model: XGE-500, made by Shenyang Seagull Technology Co., Ltd., Shenyang, China) for the cladding material. The alloy master ingot (~80 g) is placed into a quartz tube with a 1 mm diameter at its lower end. The tube is subsequently evacuated to a pressure of 10^−3^ Pa, followed by the introduction of argon gas to achieve atmospheric pressure. The alloy material within the quartz tube is then induced to melt, transforming the alloy master ingot into a liquid state. Once the pressure in the storage cylinder reaches 8 MPa, the vent valve is opened to initiate powder production.

The average diameter of powder particles was determined using a linear fitting method. Specifically, a SEM image was randomly selected, and one or multiple lines were drawn across the image to intersect as many powder particles as possible. The number of intersection points between the line(s) and particle boundaries, along with the total length of the line(s), were measured. To ensure accurate measurement of grain size, five SEM images were randomly selected for statistical analysis. The resulting data were compiled into histograms, followed by Gaussian fitting to generate a fitting curve. The final average grain size was then calculated based on the fitting data.

The laser cladding tests were conducted using RFL-A2000D semiconductor fiber-coupled laser cladding equipment (Raycus, Wuhan, China) with the Q235 alloy as the substrate. It is mainly composed of a PLC control cabinet, Yaskawa six-axis mechanical arm, Swiss Raytools coaxial powder feeding laser melting head, powder feeding device, cooling machine, voltage regulator, et al. Before cladding, the surface underwent oil removal, rust removal, and rough activation treatments. The resulting HEA coatings were designated as Si0, Si4, and Si8. To ensure a dense texture and good surface formability of the cladding layer, appropriate laser cladding parameters were selected. Key process parameters included laser power (1.5 kW), cladding speed (6 mm/s), spot diameter (3.5 mm), bonding rate (50%), powder feeding speed (10 g/min), and powder feeding mode (coaxial feed). The specific parameters used in this study are listed in Table 1. During the cladding test, the cladding direction along the Y-axis had a moving distance of 30 mm, while the X-axis had a moving distance of 1.75 mm. The resulting cladding layer measured 30 mm in length and 10 mm in width.

### 2.2. Analysis and Characterization Methods

The microstructure of the laser-cladded layers was analyzed using an OLYMPUS OLS4100 metallographic microscope (Shinjuku City, Tokyo). X-ray diffraction (XRD: Bruker, Billerica, MA, USA, D8 Advance) was used to examine the Si0, Si4, and Si8 MEA powders and cladding layers. The cross-section hardness of alloy coating was tested by a microhardness tester (Huayin, Xinxiang, China, HV-1000A) with an experimental load of 100 gf and load retention time of 10 s. The experimental load was 100 gf and the load retention time was 10 s. The corrosion resistance of the HEA cladding layers in 3.5 wt.% NaCl solution was tested and analyzed using an electrochemical workstation (Shanghai Chenhua, Shanghai, China, CHI660E). In the electrochemical tests, a silver chloride electrode, the sample surface, and a platinum electrode were used as the reference electrode, working electrode, and auxiliary electrode, respectively. The scanning potential ranged from −0.6 to 0.6 V, with a scanning rate of 1 mV/s. Electrochemical impedance spectroscopy tests were conducted at frequencies ranging from 100 kHz to 100 MHz. The morphology of the coating surface and cross-section was observed using a dual-beam scanning electron microscope (SEM, ThermoFisher, Waltham, MA, USA, Helios G4 CX) equipped with an energy-dispersive spectrometer (EDS) and electron backscatter diffraction (EBSD). The surface potential of the coating was analyzed using a Kelvin probe force microscope (KPFM, Bruker: Dimension Icon) with a PFQNE-AL probe. Two types of images were obtained during the Kelvin probe analysis: sample topography and the corresponding potential image. The resulting surface potential (V_CPD_) is defined as the difference between the probe potential (V_Tip_) and the sample potential (V_Sample_): V_CPD_ = V_Sample_ − V_Tip_. The sample potential is inversely proportional to the sample’s work function. Lower potential values correspond to a higher contact potential, greater work function, and lower surface activity.

## 3. Results and Discussion

### 3.1. Microstructure and Phase Analysis of the Powder

**Figure 1:**
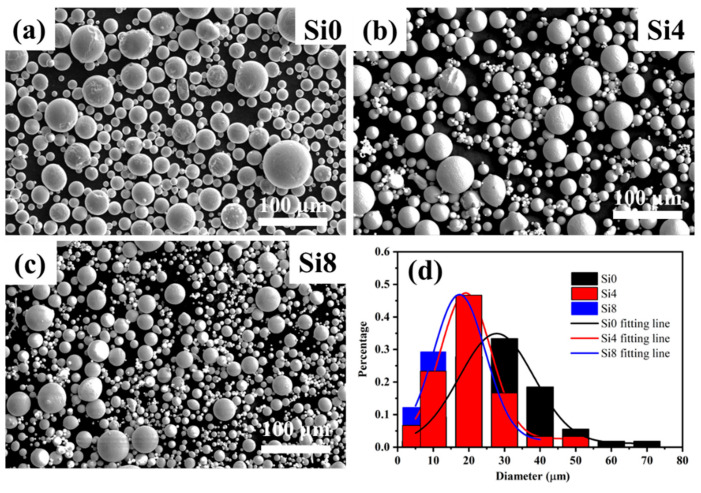
SEM images of (**a**) Si0, (**b**) Si4, and (**c**) Si8 high-entropy powders, along with (**d**) grain size distribution.

The SEM morphologies of Si0, Si4, and Si8 HEA powders are shown in Figure 1. The powders exhibit good sphericity, with only a small proportion of dumbbell-shaped and irregularly shaped particles. Such morphology can reduce friction resistance between particles and enhance powder fluidity, facilitating uniform delivery during the laser cladding process. The average grain sizes of Si0, Si4, and Si8 powders are 28 μm, 19 μm, and 17 μm, respectively. This indicates that the addition of Si improves alloy fluidity and results in smaller particle sizes in the high-entropy alloy powders.

**Figure 2:**
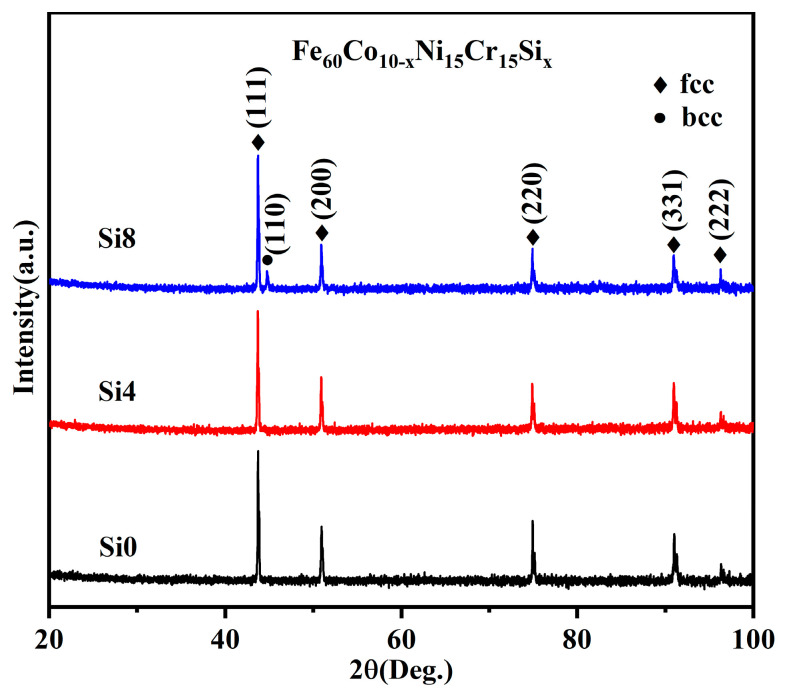
XRD patterns of Si0, Si4, and Si8 powders.

As shown in Figure 2, single FCC solid solution phases are exclusively observed in alloys with molar Si ratios of zero and four. However, with increased Si content, a BCC phase is gradually formed in the Si8 high-entropy power. This suggests that the addition of Si promotes the formation of the BCC phase in the alloy system [33].

### 3.2. Microstructure and Structural Analysis of the Coating

**Figure 3:**
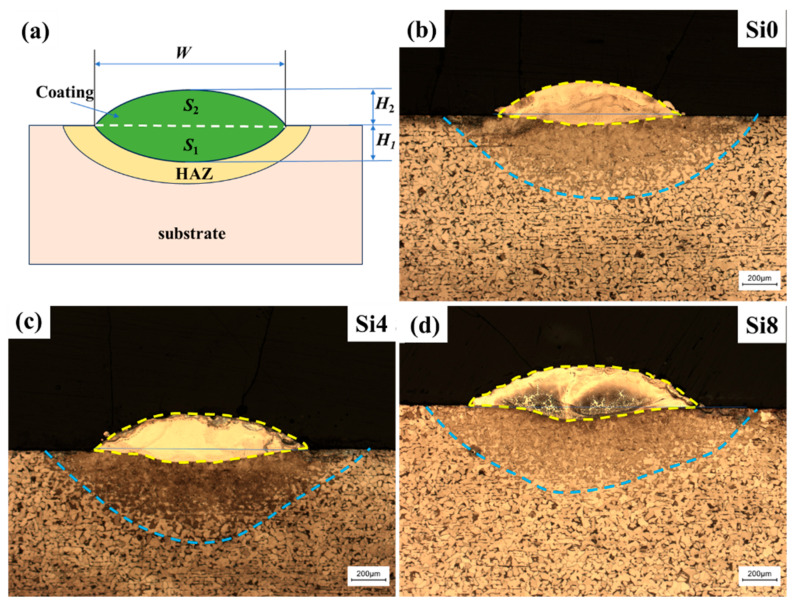
(**a**) Simplified profile of the coating and optical cross-sectional profiles of (**b**) Si0, (**c**) Si4, and (**d**) Si8 HEA coatings with a one-way path.

Figure 3a shows the cross-sectional macroscopic morphology of the one-way laser-cladded sample. The sample can be divided into three regions: the coating, the heat-affected zone (HAZ), and the substrate. The coating’s arched surface is positioned above the substrate, with a clearly visible fusion line between the coating and the HAZ, indicating a good metallurgical bond. The coating is dense and free of defects such as cracks and pores. Variations in dilution rates are evident, and the dilution rate (η) can be calculated using the following formula [34,35]:

(1) $$\eta =\frac{S_{1}}{S_{1}+S_{2}}\times 100\%\;$$

where *S*_1_ is the matrix melting area, and *S*_2_ is the coating area above the matrix surface. The coating cross-section can be modeled as two ideal arcs with different radii, as shown in Figure 3a. Based on the measured values of the thickness of the cladding layer (*H*_1_), the thickness of the laser penetration layer (*H*_2_), and the width of the coating (*W*) in Figure 3b–d, *S*_1_ and *S*_2_ can be calculated:

(2) $$S_{1}={\left\{\frac{\left[{\left(\frac{W}{2}\right)}^{2}+H_{1}^{2}\right]}{2H_{1}}\right\}}^{2}\times \left(\frac{2\pi }{360}\right){Sin}^{-1}\left\{\frac{W\cdot H_{1}}{\left[H_{1}^{2}+{\left(\frac{W}{2}\right)}^{2}\right]}\right\}-\frac{W\left[{\left(\frac{W}{2}\right)}^{2}-H_{1}^{2}\right]}{4H_{1}}$$

(3) $$S_{1}={\left\{\frac{\left[{\left(\frac{W}{2}\right)}^{2}+H_{2}^{2}\right]}{2H_{2}}\right\}}^{2}\times \left(\frac{2\pi }{360}\right){Sin}^{-1}\left\{\frac{W\cdot H_{2}}{\left[H_{2}^{2}+{\left(\frac{W}{2}\right)}^{2}\right]}\right\}-\frac{W\left[{\left(\frac{W}{2}\right)}^{2}-H_{2}^{2}\right]}{4H_{2}}$$

The thickness of the coatings is about 250 μm, with no observable defects such as macroscopic cracks, porosity, or unmelted powder in the cladding zone. As shown in Figure 3b–d, the HEA coating can be divided into four regions along the cross-section: coating, HAZ, transition zone (TZ), and substrate. The transition zone confirms the formation of a good metallurgical bond between the alloy coating and substrate using laser cladding technology. As the Si content increases, the dilution rate also increases (28.8% for Si0, 31.5% for Si4, and 34.4% for Si8). The dilution rate primarily depends on the energy absorbed by the substrate, which is closely related to the energy absorbed by the prior layer. Since the process parameters, as well as the thickness, density, and surface roughness of the cladding layer, are nearly identical in this study, the absorbed energy is mainly influenced by the chemical composition of the cladding layer. The change in dilution rate with Si content results from the interaction of the above factors. The experimental results suggest that high absorptivity compensates for the adverse effect of increased energy on substrate melting. Specifically, when x is increased to four, the high absorptivity due to increased Si content outweighs the increase in dilution rate.

**Figure 4:**
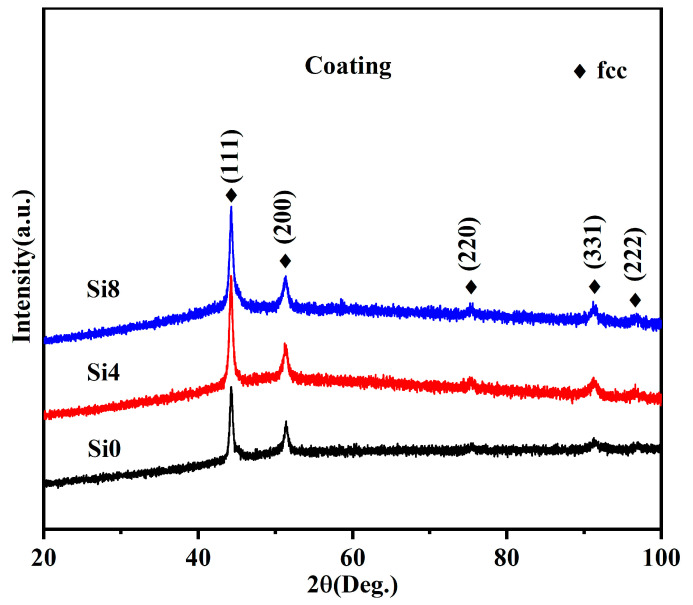
XRD patterns of Si0, Si4, and Si8 high-entropy coatings.

Si was added to the FeCoCrNi HEA system to maintain its single-phase FCC solid solution structure without forming secondary or precipitate phases. A single-phase FCC solid solution can only exist in the FeCoCrNi system when the Si content is below 5.88 at.% [24]. Figure 4 shows the XRD pattern of the HEA coatings. The results confirm an FCC structure after laser cladding, with no evident BCC peaks, indicating that the coatings derived from the three alloy powders are FCC structure. During the formation of high-entropy alloys, the phase development is closely associated with the cooling rate. In general, a high cooling rate facilitates the promotion of martensitic transformation [36]. The discrepancies in the XRD results between the powder (see Figure 2) and the coating are similar to results of our previous studies [37]. This suggests that the cooling rate exerts a substantial influence on the microstructure of FeCoCrNiSi high-entropy alloys during the preparation process.

**Figure 5:**
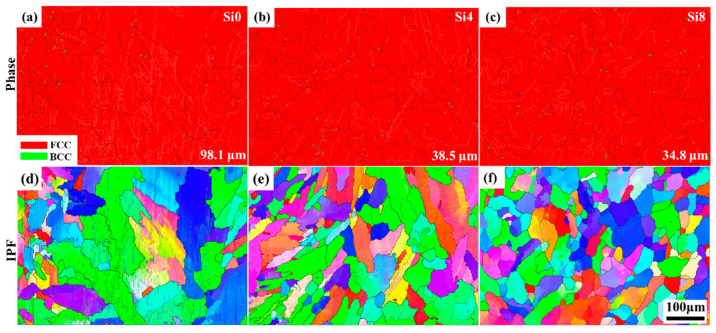
Phase map and inverse pole figure of the surface of (**a**,**d**) Si0, (**b**,**e**) Si4, and (**c**,**f**) Si8 HEA coatings; the average grain size is shown in (**a**–**c**).

To further analyze the microstructure and grain orientation of the cladding layer after LC, EBSD analysis was performed on the surface of Si0, Si4, and Si8 MEA coatings. As shown in Figure 5a–c, the surface structures of all coatings exhibit FCC symmetry. Additionally, the grain sizes of the Si0, Si4, and Si8 coatings were measured to be 98.1 μm, 38.5 μm, and 34.8 μm, respectively. The addition of Si enhances grain growth potential in the high-entropy coating. As grains grow, the number of grain boundaries decreases, reducing grain boundary corrosion during the corrosion process and improving the coating’s corrosion resistance.

**Figure 6:**
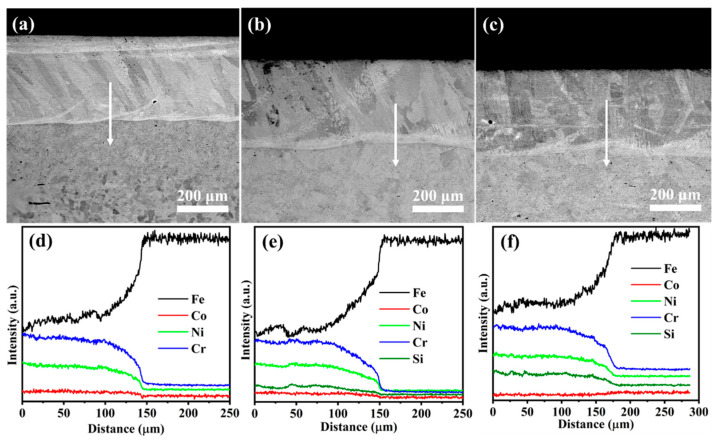
Cross-sectional SEM images and energy-dispersive X-ray spectroscopy results for (**a**,**d**) Si0, (**b**,**e**) Si4, and (**c**,**f**) Si8 high-entropy alloy coatings.

The cross-sectional morphologies from the coating to the substrate for Si0, Si4, and Si8 coatings are shown in Figure 6, revealing an approximate coating thickness of 350 μm (Figure 6a–c). No visible cracks, pores, or other defects were observed, which is consistent with the results of Figure 3 and further confirms the high quality of the prepared coatings. The TZ width is approximately 10 μm (Figure 6), indicating the formation of a metallurgical bond between the MEA coating and the Q235 steel substrate via laser cladding. EDS line scans in Figure 6d–f confirm a compact bonding interface between the coating and substrate. Near the interface, planar crystal structures transition into cellular crystals as one moves toward the center of the melting pool. Element distributions along the EDS scan line, indicated by the red arrow, are shown in Figure 6d–f. Iron (Fe) content is higher near the interface (at a distance of 45–50 μm), likely due to the elevated temperature during laser cladding, which facilitates Fe diffusion from the Q235 steel into the coating. Minor amounts of Ni, Cr, and Si were detected in the substrate, indicating slight diffusion from the coating into the substrate. This interdiffusion further confirms the strong metallurgical bond formed between the MEA coating and the Q235 steel substrate.

**Figure 7:**
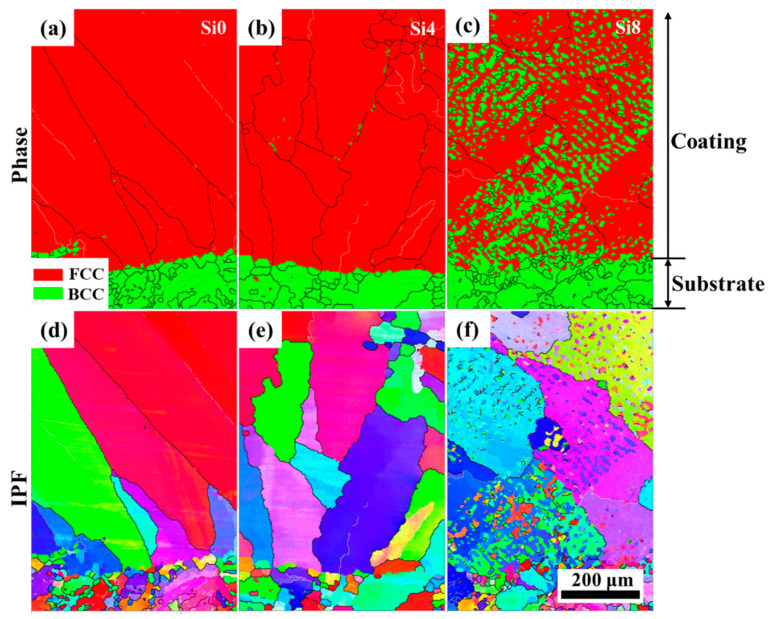
Phase images and inverse pole figures from EBSD analysis of the cross-sections of Si0, Si4, and Si8 coatings, phase of (**a**) Si0, (**b**) Si4 and (**c**) Si8 coatings, IPF of (**d**) Si0, (**e**) Si4 and (**f**) Si8 coatings.

The layered structure formed after laser cladding plays a crucial role in the fatigue resistance and strength of the cladding layer. Phase images and inverse pole figures from EBSD analysis of the cross-sections of Si0, Si4, and Si8 coatings are shown in Figure 7. The cross-sections of the Si0, Si4, and Si8 coatings exhibit a dense, well-bonded structure, characteristic of a typical metallurgical interface. The lower region of the cross-section primarily consists of coarse grains, while fine grains are observed at the joint regions. Coarse grains reappear near the transition zones. For the Si4 coating, a small amount of the BCC phase is precipitated at the boundaries of the coarse grains. In the Si8 coating, the BCC structure is more prominently observed within both the grain boundaries and interiors. This indicates that increasing the Si content enhances the powder’s fluidity during melting, leading to improved bonding with the substrate.

### 3.3. Microhardness

**Figure 8:**
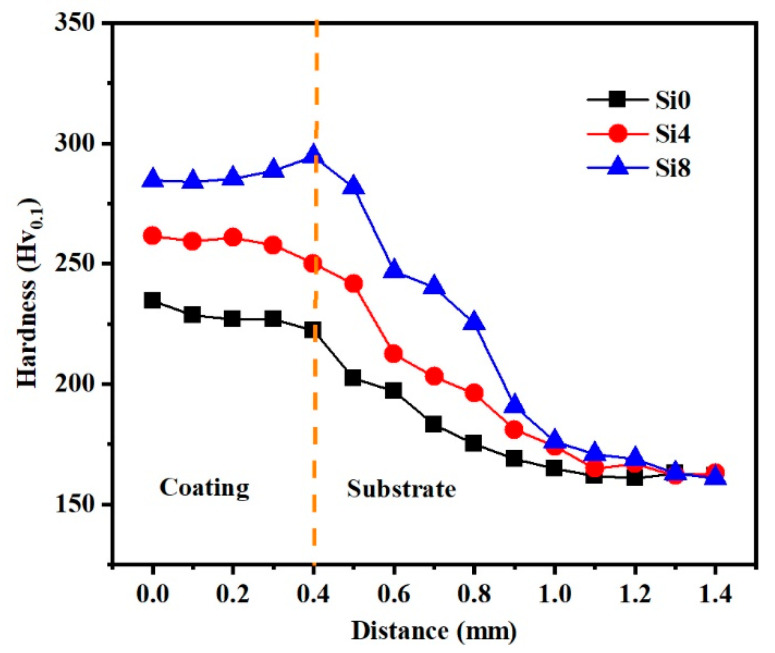
Variations in the hardness of Si0, Si4 and Si8 coatings along an increasing distance from the coating to the Q235 steel substrate.

To elucidate the mechanical behavior of the HEA coating, the hardness variation in Si0, Si4, and Si8 coatings across the cross-sectional area from the surface to the substrate is illustrated in Figure 8. It is evident that the average hardness values for the coatings are 229 Hv (Si0), 259 Hv (Si4), and 285 Hv (Si8), which are significantly higher than those of the substrate (162 Hv). The elevated hardness of the coating can be attributed to several factors: firstly, the relatively small atomic radius of silicon (Si) results in a minimal BCC phase when its content is low (≤4), with the coating’s phase structure primarily being FCC. As the Si content increases (up to Si8), Si atoms dissolve into the interstitial sites of the FCC phase, leading to an increase in both the BCC phase content and solid solubility. The incorporation of Si partially displaces other metal elements, and the difference in atomic radii enhances lattice distortion, thereby significantly increasing the solid solution strengthening effect and, consequently, the hardness. Notably, the hardness of the coating near the heat-affected zone (HAZ) increases due to the substantial formation of the BCC phase upon fusion with the substrate in the HAZ, where the hardness of the BCC phase surpasses that of the FCC phase, resulting in a gradual increase in hardness from the surface to the bonding zone. Additionally, the grain refinement induced by the rapid solidification during laser cladding, as demonstrated in Figure 5, contributes to this phenomenon.

### 3.4. Corrosion Behaviors

**Figure 9:**
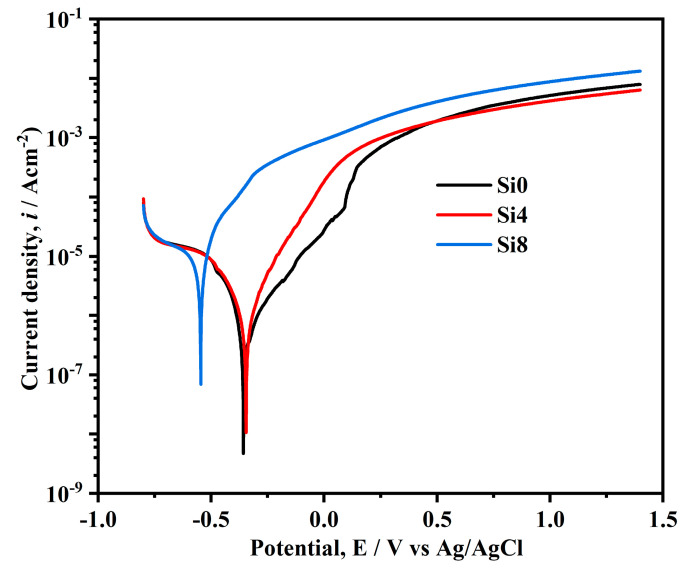
Tafel curves of Si0, Si4, and Si8 coatings in 3.5 wt.% NaCl solution.

Figure 9 illustrates the polarization curves of the Si0, Si4, and Si8 coating surfaces in a 3.5 wt.% NaCl solution. The self-corrosion potentials of Si0, Si4, and Si8 coatings are −148 mV, −157 mV, and −0.347 mV, respectively. While the self-corrosion potentials of Si0 and Si4 coatings are relatively close, the increase in Si content to eight leads to a sharp decrease in the self-corrosion potential. The Si0 coating exhibits a zigzag fluctuation in the passivation region, which is attributed to the breakdown and re-passivation of the passive film, causing pitting corrosion [38,39]. In contrast, Si8 does not display passivation in the NaCl solution, indicating poor corrosion resistance. However, Si0 and Si4 coatings show clear passivation regions with high pitting potentials, highlighting the improved corrosion resistance due to Si addition [40]. The reduced corrosion resistance of the Si8 coating may be due to Si enrichment at grain boundaries, potentially reducing intergranular corrosion of the coating. It is noted that the Si addition can cause grain refinement and increase the number of grain boundaries, as well as introduce defects during crystallization [33,41,42]. Therefore, with the addition of Si elements, the grain size of the Si8 coating decreases obviously and thus a more pronounced enrichment may occur, finally leading to decreased corrosion resistance. When the Si content in the alloy is low, the Si4 coating does not achieve the necessary concentration to mitigate corrosion, and thus the corrosion performance of the alloy remains unaffected. Consequently, the grain boundaries become preferential sites for corrosion, resulting in the poorest corrosion performance for the Si8 coating.

**Figure 10:**
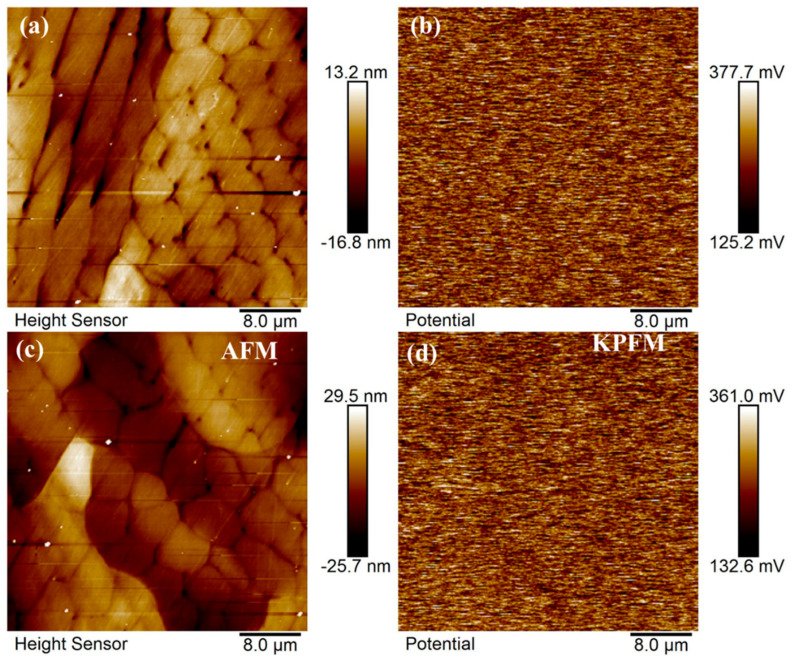
Surface morphology and corresponding KPFM images of (**a**,**b**) Si0 and (**c**,**d**) Si4 coatings.

Due to the similar corrosion resistance of Si0 and Si4 coatings, surface potential analysis was performed. The surface morphology and contact potential (V_CPD_) of Si0 and Si4 coatings, characterized using Kelvin probe force microscopy, are shown in Figure 10. Results indicate that lower V_CPD_ corresponds to a higher work function, reduced surface activity, and greater corrosion resistance [43]. Figure 10a,c reveal that as Si content increases, the coating surface transitions from dendritic to equiaxed crystals, with a reduction in grain size [42,44]. Figure 10b,d show minimal potential differences between grains and grain boundaries, as well as between strip and dendritic crystals on the surfaces of Si4 and Si8 coatings. XRD and EBSD results confirm that the coating surfaces predominantly consist of a single FCC phase, resulting in a uniform alloy composition. This uniformity minimizes potential differences and reduces the influence of alloy composition on the corrosion process.

**Figure 11:**
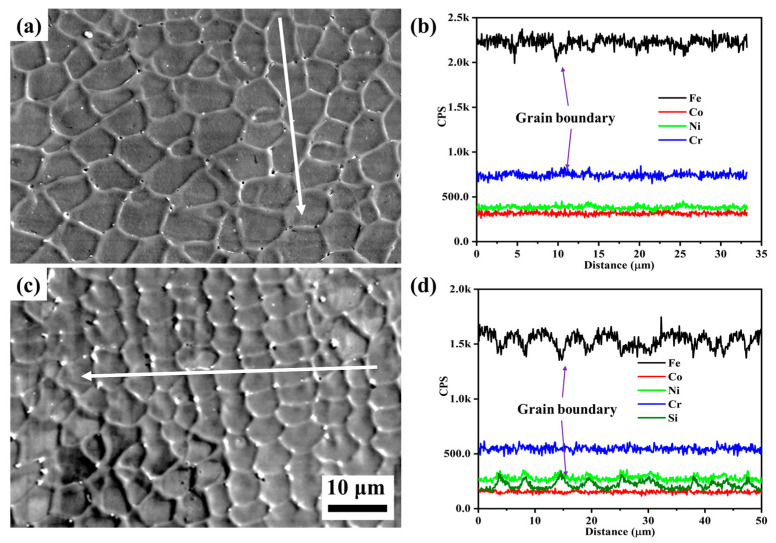
Secondary electron images and line scan results of (**a**,**b**) Si0 and (**c**,**d**) Si4 coatings after corrosion tests in 3.5 wt.% NaCl solution.

To further clarify the differences in corrosion behavior between Si0 and Si4 coatings, post-corrosion morphology and the corresponding line scan results are presented in Figure 11. Both coatings exhibit equiaxed crystal structures, with Si4 having smaller grains. After corrosion, small pitting pits appear at the grain boundaries of both coatings. Elemental analysis (Figure 11b,d) shows a reduction in Fe content and an enrichment of Cr and Ni elements at the grain boundaries of the Si0 alloy. In the Si4 coating, Si, Cr, and Ni element are enriched at the grain boundaries, suggesting that Si addition promotes Cr and Ni segregation in this region. Corrosion typically initiates at grain boundaries, where Cr and Si form oxide films such as Cr_2_O_3_ and SiO_2_. As these oxides grow, thermal stresses can cause cracking and spallation, exposing fresh metal [41]. However, the presence of SiO_2_ in the passivation film may enhance the stability of Cr_2_O_3_, improving the corrosion resistance of the Si4 coating [30].

## 4. Conclusions

This study explored the effect of Si addition on the microstructure and corrosion resistance of Fe_60_Co_10−x_Ni_15_Cr_15_Si_x_ (x = 0, 4, and 8) high-entropy alloy coatings. High-entropy alloy powder was synthesized via aerosol technology, and coatings were prepared using laser cladding. The main findings are as follows:

1. Si addition enhances the powder’s fluidity during melting, leading to improved bonding with the substrate.
2. Si addition leads to its enrichment at grain boundaries, hindering grain growth and refining the grain size of the coating.
3. For the studied coatings, all samples exhibit an FCC structure, with only minor BCC phases at grain boundaries. At the coating–substrate interface, increasing Si content results in a transition from a single-phase FCC to an FCC + BCC structure.
4. The Si4 coating demonstrates superior corrosion resistance, attributed to the Si-enhanced enrichment of corrosion-resistant elements at grain boundaries. This enrichment stabilizes the passivation film, effectively improving corrosion resistance while maintaining low production costs.

## Figures and Tables

**Table 1:** Composition of the Q235 alloy.

Elements	C	Si	Mn	S	P	Fe
**Content (wt.%)**	0.150	0.200	0.460	0.022	0.012	Balance

**Table 2:** The purity, shape, and size of high-purity Fe, Co, Ni, Cr, and Si materials for this study.

Element	Purity (wt.%)	Shape	Size
Fe	99.9%	Block	3 × 3 mm
Co	99.95%	Block	5–10 mm
Ni	99.9%	Block	3 × 3 mm
Cr	99.95%	Block	1–5 mm
Si	99.999%	Block	1–6 mm

## Data Availability

The original contributions presented in this study are included in the article. Further inquiries can be directed to the corresponding authors.
